# Connectivity expectations as psychological contract terms in the digital workplace

**DOI:** 10.3389/fpsyg.2026.1852486

**Published:** 2026-06-04

**Authors:** Nthabeleng Innocentia Mdhluli

**Affiliations:** Industrial and Organisational Psychology, University of South Africa, Pretoria, South Africa

**Keywords:** after-hours communication, boundary management, digital breach, digital connectivity, digital work, organisational trust, psychological contract, technostress

## Abstract

**Introduction:**

The digitalisation of work has normalised expectations of continuous employee availability, yet the relational and contractual implications of these expectations remain insufficiently theorised. This conceptual article contends that connectivity expectations constitute an implicit domain of psychological contract obligation. The perceived violation of this obligation, termed digital breach, differs phenomenologically from traditional breach. Its distinctness arises from technological mediation, which transforms personal availability from a private relational gesture into a monitored, metric-visible professional signal.

**Methods:**

Drawing on psychological contract theory, digital communication research, boundary management research, and technostress literature, the study develops the Digital Psychological Contract Model (DPCM).

**Results:**

The DPCM specifies a propositional architecture that links connectivity expectations and enacted availability behaviours, through discrepancy assessment, to digital breach, affective violation, and the erosion of organisational trust. Perceived autonomy and leadership norm-modelling enter as moderating conditions; organisational communication culture and leadership signalling enter as antecedents.

**Discussion:**

The model provides a conceptual scaffold and structured vocabulary for an emerging research programme on psychological contracts in digitally mediated work. It also differentiates digital breach from technostress, organisational injustice, and traditional contract breach, and identifies measurable indicator domains for future scale development.

## 1 Introduction

The digitalisation of work has fundamentally altered the temporal and relational architecture of contemporary employment. Communication technologies that once provided flexibility and autonomy have become integrated into organisational routines. They now shape expectations for how, when, and how quickly employees must respond to work demands. Early sociological studies observed that mobile technologies blur work and non-work distinctions by extending organisational presence into employees’ personal time (Wajcman and Rose, 2011). More recent research indicates that these dynamics have accelerated significantly. As digital infrastructures became more central to coordination and performance management, previously exceptional forms of constant connectivity became routine (Eurofound, 2021; Messenger et al., 2017). The rapid acceleration of hybrid and remote working arrangements during and after the COVID-19 pandemic has reinforced these expectations, with new ways of working resulting in both flexibility and increased pressure to remain digitally available across time boundaries (Alfes et al., 2022).

The implications of such developments in industrial and organisational psychology have primarily been examined through the lens of work stress and recovery. Empirical research indicates that after-hours digital communication can disrupt psychological detachment from work. Detachment is widely acknowledged as necessary for restoring depleted cognitive and emotional resources (Barber et al., 2019; Sonnentag, 2012; Sonnentag and Fritz, 2015). The technostress literature demonstrates how persistent digital demands contribute to overload, perceived invasiveness, and role ambiguity, all of which impair well-being and job satisfaction (Ayyagari et al., 2011; Tarafdar et al., 2019). These effects have been amplified in remote and hybrid work contexts where digital platform-induced stress is compounded by the blurring of physical and temporal work boundaries (Singh et al., 2022; Van Zoonen et al., 2023). These perspectives provide valuable insight into strain. What they do not capture is the relational meaning of connectivity expectations within the employment relationship. Both frameworks view availability as a task-level demand, rather than a signal embedded in the employment relationship.

Psychological contract theory offers a different perspective. Rousseau's (1995) foundational work defined psychological contracts as subjective beliefs about mutual obligations. Subsequent research has established that these beliefs are influenced not only by formal agreements, but also by organisational practices, leadership behaviour, and informal norms (Conway and Briner, 2005; Guest, 1998; Rousseau et al., 2018). When employees believe that the organisation has failed to meet implied obligations, they may experience contract breach. In more affectively intense circumstances, they may experience contract violation. These reactions can erode trust and weaken commitment (Morrison and Robinson, 1997; Robinson and Morrison, 2000; Zhao et al., 2007). A systematic review of meta-analyses on psychological contract breach confirms that these relationships exist across contexts, although moderated by individual and situational factors (Topa et al., 2022). Despite the theory’s continued relevance, the digital transformation of work has resulted in new forms of implicit expectation that have not been adequately theorized within this framework.

Recent scholarship has begun to map the topic empirically. Zhang et al. (2023) demonstrated that after-hours electronic communication expectations were associated with psychological contract breach among nursing professionals, predicting withdrawal through this relational pathway. Ballas et al. (2024) identified digital communication norms as a new dimension reshaping psychological contracts in digitally mediated workplaces. These advancements are valuable. What remains absent, however, is a conceptual framework that specifies digital availability as a distinct contractual domain, what characteristics distinguish its violations from traditional breaches, and how such violations undermine organisational trust. There is a theoretical and construct-level gap that this article directly addresses.

The contribution of this article is conceptual rather than empirical or causal. It offers a structured vocabulary and conceptual scaffold for an emerging research programme on psychological contracts in digitally mediated work. Specifically, the article integrates psychological contract theory with research on digital work practices, boundary management, and technology-enabled work intensification to develop the Digital Psychological Contract Model (DPCM). The model articulates how connectivity expectations may constitute implicit contractual terms and how their interaction with enacted availability patterns may produce a perception of digital breach. Additionally, the model explains how digital breach may translate into affective contract violation under specific conditions and how this pathway may erode the integrity-based and benevolence-based dimensions of organisational trust. The propositions presented are research-generative rather than tested causal claims. They define a propositional architecture that future empirical work may refine, qualify, or revise.

The article proceeds as follows. Section 2 describes the conceptual approach and methodological foundations. Section 3 reviews the relevant literatures and positions the study’s theoretical contribution. Section 4 presents the DPCM and its constituent propositions. Section 5 discusses theoretical and practical implications, limitations, and directions for future research. Throughout, the article contributes to ongoing scholarship on the human consequences of digital transformation and highlights the importance of intentional governance of digital availability norms in sustaining healthy employment relationships.

## 2 Conceptual approach and methodological foundations

This study adopts a conceptual integration approach (Jaakkola, 2020), appropriate for developing new theoretical frameworks by synthesising and reinterpreting existing bodies of knowledge. Specifically, the study follows Jaakkola’s (2020) model-building approach, which necessitates three deliverables: specification of the model’s scope conditions, explanation of the theoretical logic connecting model components, and identification of construct measurement implications. The scope conditions of the DPCM are formally specified in Section 4.6. The propositional logic is developed in Sections 4.1–4.5. The measurement implications of the digital breach construct are discussed in Section 5.5. Following Jaakkola’s explicit guidance that the literature corpus of a conceptual paper functions as the equivalent of an empirical sample, this section also documents the corpus, its composition, and the synthesis process. The full literature corpus is reported in Appendix A.

### 2.1 Literature identification and selection

The literature base was constructed through structured purposive searching across three primary theoretical domains: (1) psychological contract theory, (2) digital work and technostress, and (3) boundary management. Two cross-cutting domains, organisational trust and methodological or foundational sources were retained for theoretical anchoring. Search platforms were APA PsycINFO, Web of Science, and Google Scholar. Search terms included “psychological contract,” “psychological contract breach,” “psychological contract violation,” “digital work,” “after-hours communication,” “technostress,” “boundary management,” “work-home boundary,” “remote work,” “hybrid work,” and “always-on.” The search was conducted between June and October 2024.

The initial database retrieval yielded approximately 280 records. Title and abstract screening for relevance to the construct space (digital availability expectations, psychological contract dynamics in technologically mediated work, and the boundary management of digital communication) reduced the corpus to 142 records. Full-text screening removed sources that were unrelated, methodologically weak, or duplicated by stronger sources within the same finding cluster. The final corpus used for conceptual integration consists of 62 sources, which are listed in full in Appendix A (Supplementary Table A1) and depicted in the review process flow diagram (Supplementary Figure A1).

Corpus composition is as follows. By domain (with some sources spanning multiple domains), the following are the number of sources: psychological contract theory (18 sources), digital work and technostress (27 sources), boundary management (14 sources), organisational trust (4 sources), and methodological/foundational (5 sources). Empirical studies accounted for 28 sources (45%), theoretical and conceptual works for 20 (32%), and reviews and meta-analyses for 14 (23%). By date: 24 sources (39%) were published since 2020, addressing post-pandemic digital work dynamics; the remaining 38 sources (61%) are foundational theoretical works or pre-pandemic empirical evidence retained for construct grounding.

The synthesis adhered to a theory adaptation logic (Jaakkola, 2020). The established psychological contract concepts of breach, violation, and trust were extended into the newly developed context of digitally mediated work practices through a systematic comparison with the digital work and boundary management literatures. Where established constructs were sufficient, the synthesis preserved them. Where the digital context introduced features unaccommodated by existing constructs, the synthesis articulated extensions. This comparative process resulted in the five-dimensional construct of digital breach (Section 4.2); Section A.2 of the Appendix details how each dimension was explicitly derived from specific source clusters.

### 2.2 Construct development process

The concept of digital breach was developed iteratively by identifying differences between traditional breach contexts and digitally mediated work environments. A comparative analysis of the digital work and boundary management literatures revealed five dimensions where digital availability expectations differ from traditional organisational obligations: (1) diffuse temporality, (2) implicit formation, (3) technological mediation, (4) normalised pervasiveness, and (5) continuous negotiation. These dimensions were synthesized into a working definition of digital breach as the perceived discrepancy between expected and enacted patterns of digital availability within the employment relationship. The construct was then refined through repeated comparison with the broader psychological contract literature to ensure conceptual coherence and discriminant distinctiveness. Each of the five dimensions can be traced to specific source clusters within the corpus, as documented in Appendix A.2.

### 2.3 Proposition derivation

Propositions were derived from the conceptual model architecture. Following Whetten's (1989) guidelines, each proposition specifies the nature, direction, and conditional logic of the proposed relationship, as well as the underlying mechanism that makes the relationship theoretically necessary. The propositions are presented as research-generated rather than tested causal claims. They define a propositional architecture that future empirical work may refine, qualify, or revise. Propositions 1–3 describe the core mediation pathway that links connectivity expectations to trust erosion through discrepancy assessment, digital breach, and affective violation. Propositions 4a and 4b describe moderating conditions derived from the boundary management and leadership literatures. Proposition 5 specifies the antecedent organisational conditions that lead to connectivity expectations.

### 2.4 Limitations of the approach

Consistent with the aims of conceptual integration, this study does not claim empirical testing of the proposed relationships. The framework is a generative foundation for future empirical investigation. The literature corpus, although structured and documented, is not exhaustive. The synthesised literature is drawn predominantly from Western, English-language scholarship, which reflects an inherent geographic and cultural bias. Connectivity norms, boundary management expectations, and employment relationship dynamics can vary significantly across institutional, cultural, and collectivist work settings (Ollier-Malaterre and Foucreault, 2017). Alternative literatures, such as labour process theory and algorithmic management scholarship, may provide complementary perspectives that are not fully integrated here. These limitations are discussed further in the Discussion section.

## 3 Literature review

### 3.1 Digitalisation, connectivity, and the temporal reorganisation of work

The expansion of digital communication technologies has reconfigured not only where work is performed but also when and how organisational expectations are enacted. Digital infrastructures enable real-time coordination across geographical and temporal boundaries, allowing organisations to maintain continuous operational responsiveness. While such developments are often associated with increased flexibility and autonomy, research suggests that they also contribute to the intensification of temporal demands and the blurring of clear work–life boundaries (Eurofound, 2021; Messenger et al., 2017). Rather than simply extending traditional work practices into new technological environments, digitalisation transforms the conventional assumptions underlying availability, responsiveness, and performance. Wajcman (2015) characterizes this as the temporal restructuring of digital capitalism, in which time pressure becomes structurally reproduced rather than individually experienced.

Empirical scholarship shows that after-hours communication has become a routine feature of many professional roles, particularly in knowledge-intensive sectors. Studies indicate that employees frequently experience implicit pressure to remain reachable through email or messaging platforms, even in the absence of explicit organisational policies (Schlachter et al., 2018). Research on technology-assisted supplemental work indicates that using work-related technology outside of contractual hours increases work-to-family conflict and reduces recovery quality. This is particularly the case when organisational expectations rather than personal choice drive the behaviour (Fenner and Renn, 2010; Towers et al., 2006). The rapid adoption of remote and hybrid work since 2020 has intensified these dynamics. Digital availability expectations have become particularly prominent in work-from-home contexts, where the absence of physical work–family separation enhances the perceived obligation to remain reachable (Afota et al., 2023; Becker et al., 2022). Research on workplace segmentation norms demonstrates that employees who perceive strong organisational expectations of availability experience greater difficulty disengaging from work-related communication, with smartphone use outside working hours predicting reduced psychological detachment and heightened exhaustion (Derks et al., 2014). Over time, these dynamics can contribute to the normalization of an “always-on” orientation in which temporal flexibility becomes accompanied by increased performance visibility and reduced opportunities for psychological recovery (Ter Hoeven et al., 2016; Van Zoonen et al., 2023).

The consequences of sustained connectivity are increasingly recognised. Research on recovery processes highlights the importance of psychological detachment for maintaining cognitive functioning and emotional stability (Sonnentag, 2012; Sonnentag and Fritz, 2015). When digital communication intrudes into non-work domains, opportunities for detachment are limited, resulting in cumulative stress and decreased work engagement. Dettmers (2017) demonstrated that extended work availability mediates the relationship between job demands and well-being through reduced psychological detachment, providing direct evidence for the recovery-disruption pathway. Additionally, Chesley (2005) demonstrated that technology-enabled boundary blurring is linked to higher levels of individual distress and decreased family satisfaction, even when work demands remain constant. This implies that the adverse effects are triggered by permeability itself, rather than the volume of work. In hybrid and fully remote work settings, digital platform-induced technostress has emerged as a particularly consequential pathway from connectivity demands to exhaustion and subjective well-being deterioration (Harunavamwe and Ward, 2022; Singh et al., 2022). The technostress literature identifies mechanisms such as information overload and technology-enabled interference that reduce perceived control and increase emotional exhaustion (Ayyagari et al., 2011; Ragu-Nathan et al., 2008; Tarafdar et al., 2019). These perspectives provide valuable insights into the demands of digital work. However, both view connectivity primarily as a functional feature of job design, rather than a relational signal embedded in the employment exchange. These demands are also unevenly distributed across the workforce. In certain organisational contexts, they may serve as performance surveillance tools, a dimension that mainstream occupational health frameworks have not fully explored.

### 3.2 Psychological contract theory and the dynamics of reciprocal obligation

Psychological contract theory provides a complementary framework for understanding how employees interpret organisational expectations and create meaning from their work relationships. Psychological contracts refer to individuals’ beliefs about mutual obligations that arise from perceived promises, organisational practices, and social interactions (Rousseau et al., 2018). These beliefs are inherently subjective and fluid, changing as employees interpret organisational actions and adjust their expectations accordingly. The construct has attracted sustained scholarly attention since Rousseau’s (1989) early theorization, with Guest (1998) arguing that psychological contracts capture dimensions of the employment relationship that formal contracts cannot. Psychological contracts go beyond traditional employment agreements. They include implicit norms for fairness, support, and behavioural expectations.

Research has consistently demonstrated that perceived contract breach, defined as the cognitive recognition that organisational obligations have not been fulfilled, can trigger emotional reactions known as contract violation (Morrison and Robinson, 1997). Such reactions are linked to decreased organisational trust, lower commitment, and negative behavioural outcomes such as withdrawal and decreased performance (Coyle-Shapiro and Kessler, 2000; Ng et al., 2010; Zhao et al., 2007). A recent systematic review of meta-analyses on psychological contract breach confirms that these relationships are robust across work contexts, with organisational trust and affective commitment consistently emerging as the most proximate outcome variables (Topa et al., 2022). Robinson and Morrison (2000) further demonstrated in a longitudinal study that breach perceptions develop gradually over time and that early breach experiences predict later violation responses, consistent with a cumulative process model. Extending this logic to digital work contexts, Zhang et al. (2023) observed that after-hours electronic communication expectations were associated with psychological contract breaches, which predicted withdrawal behaviours among nurses. This provides preliminary empirical evidence for the importance of contract dynamics in digitally mediated work. Subsequent research has emphasized the importance of organisational communication and leadership behaviour in shaping psychological contract formation, highlighting how implicit signals and informal practices shape employees’ interpretations of reciprocal exchange (Conway and Briner, 2005).

Despite the theory’s importance in understanding changing work arrangements, little attention has been given to how technological change may introduce new domains of perceived obligation. As work is increasingly mediated by digital communication systems, expectations of availability and responsiveness may gain relational significance. A recent systematic review by Ballas et al. (2024) confirms this need. They identify online communication norms and digital capabilities as emerging dimensions that fundamentally alter psychological contracts in digitally mediated work environments. They recommend the development of theoretical frameworks that can specify the mechanisms involved.

### 3.3 Boundary management and the relational meaning of connectivity

Research on boundary management provides further insight into how employees navigate competing demands across work and non-work domains. Nippert-Eng’s (1996) foundational work established that the work–home boundary is a socially constructed and actively maintained accomplishment rather than a fixed structural condition. Boundary theory subsequently formalised this insight, proposing that individuals actively construct and maintain psychological and behavioural boundaries to manage role transitions and preserve personal resources (Clark, 2000). Digital technologies complicate this process by enabling the rapid transfer of work demands into personal contexts, thereby increasing boundary permeability. Employees may respond by adopting segmentation or integration strategies depending on personal preferences and organisational expectations. They may also deploy a range of active boundary work tactics, including temporal, physical, and communicative strategies, to manage competing role pressures (Kreiner et al., 2009). Research on boundary management profiles suggests that the fit between individual preferences and organisational norms is a significant predictor of work-to-home conflict and well-being outcomes (Kossek et al., 2012). This suggests that misalignment between expected and preferred availability carries well-being costs that extend beyond mere workload considerations. Recent research has demonstrated that in remote and hybrid work contexts, constant connectivity perceptions activate boundary management behaviours, with organisational ICT policies and informal social norms serving as primary contextual determinants of how employees manage availability boundaries (Van Zoonen et al., 2023).

Boundary management research also indicates that the consequences of boundary permeability are shaped by relational and organisational factors. When availability expectations are perceived as voluntary or mutually negotiated, employees may find flexibility empowering. When connectivity demands are perceived as imposed or unfair, they can elicit resentment and feelings of exploitation (Barber and Santuzzi, 2015; Butts et al., 2015). Butts et al. (2015) reported that the emotional consequences of after-hours electronic communication depended substantially on whether employees attributed such communication to organisational or personal necessity. This is consistent with the appraisal-based logic the present model employs. The impact of digital communication on well-being cannot be fully understood without considering how employees interpret such demands within the broader relational context of employment.

### 3.4 Towards a Digital Psychological Contract Framework

The preceding literature highlights the need for an integrative perspective connecting digital work practices, well-being outcomes, and relational dynamics. It is essential to demonstrate why the psychological contract lens offers a distinct contribution compared to established frameworks.

Job Demands–Resources (JD–R) theory conceptualises connectivity as a demand that consumes energy, potentially leading to burnout, while resources such as autonomy can buffer its effects (Bakker and Demerouti, 2017; Schaufeli and Bakker, 2004). JD–R focuses primarily on the energetic process. It does not explain how employees interpret connectivity demands as signals of reciprocity, fairness, and organisational intent. A psychological contract perspective captures this interpretive layer. It explains why similar levels of connectivity demand might be experienced as a violation by one employee and as reciprocal flexibility by another.

Boundary theory focuses on how individuals manage transitions between work and non-work roles, with connectivity increasing boundary permeability (Ashforth et al., 2000). This highlights the operational challenges of digital work. It does not, however, address the relational meaning that employees attribute to these intrusions. The psychological contract lens frames boundary intrusions not just as challenges to role management but as potential breaches of implicit agreements regarding the organisation’s respect for an employee’s personal domain.

Social exchange theory argues that relationships evolve into trusting, mutually committed exchanges based on generalised reciprocity (Blau, 1964; Cropanzano and Mitchell, 2005). Psychological contract theory offers a more precise framework for understanding the cognitive and affective reactions to unmet expectations. The concepts of breach and violation provide a specific mechanism, rooted in perceived obligations, that explains why trust may be abruptly eroded. This dynamic is central to digitally mediated work where expectations are often implicit and ambiguous.

By integrating the relational precision of psychological contract theory with insights from these adjacent fields, the present study proposes a framework that explains how connectivity demands are interpreted, evaluated, and internalized within the employment relationship. What remains unspecified is the structural logic of this relational process. How do connectivity expectations translate into perceived obligations? When do discrepancies become consequential? Through what mechanisms do they influence organisational trust? The following section presents the DPCM, which formalises these questions into a coherent conceptual framework for digital availability expectations.

## 4 The Digital Psychological Contract Model

**Figure 1:**
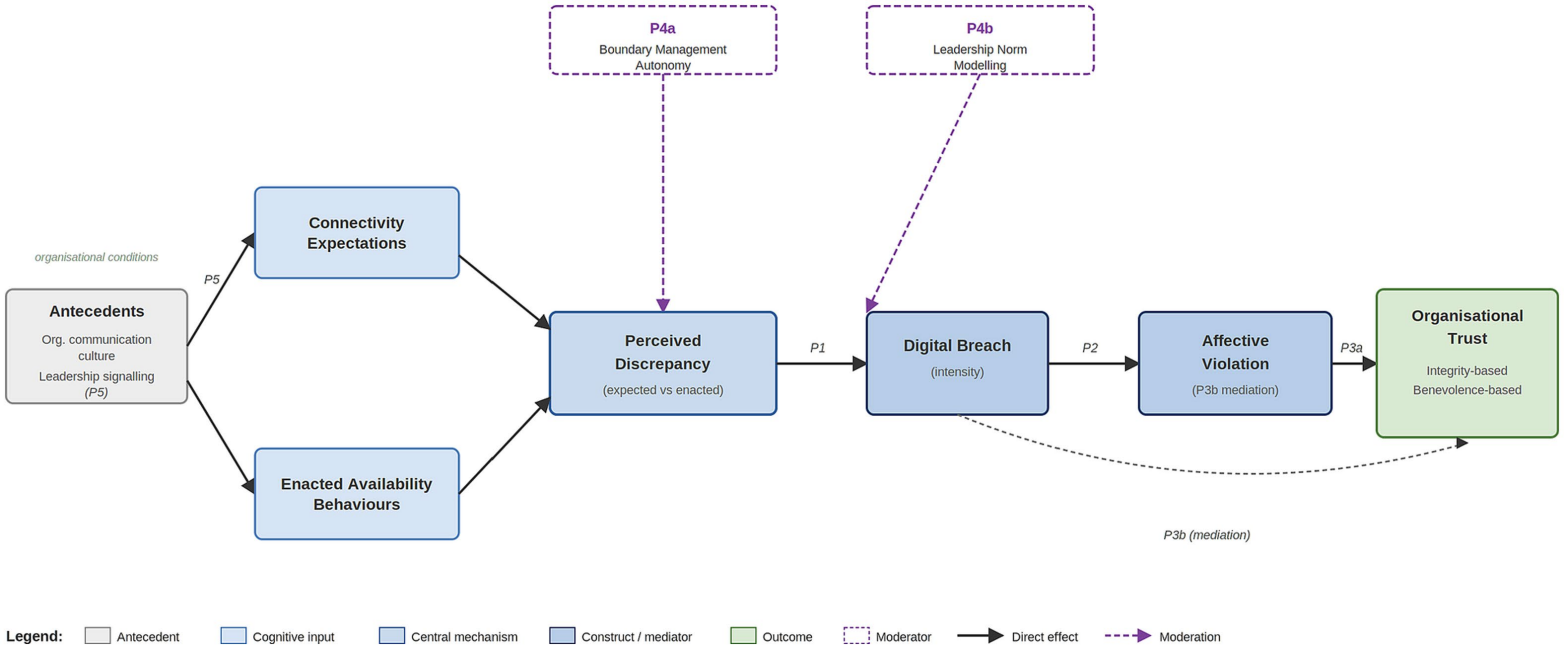
The Digital Psychological Contract Model. Connectivity expectations and enacted availability behaviors are joint inputs to a discrepancy assessment, which is the proximate predictor of digital breach intensity (P1). Digital breach predicts affective contract violation under specified moderating conditions (P2). Affective violation is negatively associated with the integrity-based and benevolence-based dimensions of organizational trust (P3a) and mediates the relationship between digital breach and trust erosion (P3b). Perceived autonomy (P4a) and leadership norm-modeling (P4b) moderate the discrepancy-to-breach path. Organizational communication culture and leadership signaling are antecedents of connectivity expectations (P5). Solid arrows represent direct propositions; dashed lines represent moderation.

Figure 1 presents the Digital Psychological Contract Model. The model articulates a propositional architecture connecting connectivity expectations and enacted availability behaviours, through a discrepancy assessment, to digital breach, affective violation, and the erosion of organisational trust. The model is structured around five propositions. P1 specifies that the magnitude of perceived discrepancy between connectivity expectations and enacted availability is the proximate predictor of digital breach intensity. P2 specifies the moderated relationship between digital breach and affective violation. P3a and P3b specify the trust outcome and its mediation through affective violation. P4a and P4b specify moderating conditions on the discrepancy-to-breach path. P5 specifies the organisational antecedents of connectivity expectations. The architecture is research-generative rather than causally tested.

### 4.1 Conceptualising connectivity expectations as contractual signals

Throughout the manuscript the term connectivity expectations is used as the dominant construct label. Where stylistic variation is necessary, connectivity expectations may be referred to as digital availability expectations or connectivity norms. These terms are treated as synonyms throughout the article, not as distinct constructs.

In digitally mediated workplaces, expectations regarding employee availability are increasingly communicated through informal organisational practices, leadership behaviour, and technological affordances rather than formal contractual agreements. Such expectations may include prompt responsiveness to email, participation in virtual meetings outside standard hours, or continuous engagement with collaborative platforms. Over time, these behavioural norms may acquire symbolic meaning, functioning as indicators of commitment, trustworthiness, and professional identity (Mazmanian et al., 2013). These expectations have been further shaped by post-pandemic shifts toward hybrid and remote work arrangements, which have redefined what “being at work” means and turned digital presence into a proxy for professional engagement (Alfes et al., 2022).

Psychological contract theory suggests that employees actively interpret organisational signals to determine reciprocal obligations within the employment relationship (Rousseau et al., 2018). From this perspective, digital connectivity expectations function as relational cues. They shape employees’ beliefs about what the organisation requires and what it will provide in return. This interpretive process is consequential. One employee may interpret the same connectivity practice as an expression of relational trust, while another may perceive it as an imposition on personal resources. The distinction lies in the relational context within which the expectation is embedded. Employees may incorporate connectivity demands into a relational psychological contract if they are perceived as legitimate and are met with support, flexibility, or recognition. When they are perceived as excessive or unfair, they can contribute to feelings of imbalance in the exchange relationship.

The proposed model conceptualises connectivity expectations as implicit relational terms embedded within contemporary employment relationships. Rather than treating after-hours communication solely as a job demand, the model positions connectivity norms as signals through which employees evaluate organisational intentions and fairness.

### 4.2 Digital breach and affective contract violation

A central construct within the model is digital breach, defined as the perceived discrepancy between expected and enacted patterns of digital availability within the employment relationship. While this builds on Morrison and Robinson’s (1997) foundational definition of breach, the digital context imbues the concept with several distinctive characteristics that warrant explicit theorization.

First, diffuse temporality: Unlike traditional breaches, which are often the result of a single event, such as a denied promotion, digital breaches are usually perceived as a cumulative process. Expectations are constantly negotiated through repeated interactions and may only be identified as unfulfilled over time.

Second, implicit formation: Connectivity expectations are rarely specified in formal agreements. They emerge from the interpretation of leadership behaviour, organisational culture, and technological affordances. This ambiguity increases the likelihood of perceptual discrepancies, as employees and employers may hold different, unspoken assumptions about appropriate responsiveness.

Third, technological mediation: The expectations themselves are mediated by technological systems that provide visibility, such as “last seen” statuses and response-time metrics. This transforms availability from a private choice into a publicly visible signal of performance, increasing the perceived obligation.

Fourth, connectivity expectations are frequently normalised as part of a “professional” identity (Mazmanian et al., 2013). This normalization can obscure their true nature as relational obligations, making perceived breaches feel like personal or professional failings rather than unmet expectations.

Fifth, continuous negotiation: The workplace relationship is no longer defined by the workday. The psychological contract regarding availability is negotiated with each after-hours email or late-night message, making it a constantly changing and potentially unstable relational concept.

These five dimensions collectively suggest that digital breach operates on a range of accumulating discrepancy rather than as a single event. The construct is therefore best understood as varying in intensity rather than being present or absent.

Technological mediation is not simply one of five parallel characteristics; it is the condition that makes the other four analytically distinctive. Through technological mediation, diffuse temporality is continuously monitored rather than episodically recalled. Response-time logs and engagement analytics provide skewed visibility into implicit formation. Normalised pervasiveness acquires professional identity formation through platform design and organisational digital culture. Continuous negotiation takes place through metric-legible performance signals rather than informal social exchange. Remove technological mediation from the framework, and what remains is a description of any gradually accumulating informal obligation. Such obligations are common in employment relationships, but not unique enough to warrant a new construct. When technological mediation is retained, digital breach becomes analytically distinguishable from traditional forms of psychological contract breach.

*Proposition 1*: Digital breach intensity is positively associated with the perceived magnitude of discrepancy between connectivity expectations and enacted patterns of digital availability within the employment relationship.

#### 4.2.1 Discriminant boundaries of the construct

Establishing the theoretical distinctiveness of digital breach requires clarity about what the construct is not. First, digital breach is not reducible to technostress. Technostress describes a strain appraisal triggered by technology-related demands, particularly overload, invasiveness, and complexity (Ayyagari et al., 2011; Tarafdar et al., 2019). Digital breach is a relational appraisal, that is, a cognitive-evaluative judgement that implicit contractual obligations regarding availability have gone unmet. Two employees experiencing identical volumes of after-hours communication may differ substantially in whether they experience digital breach. This depends on how each interprets the organisation’s implicit availability obligation. Technostress predicts stress for both, whereas digital breach logic predicts violation only for the employee who perceives an obligation gap.

Second, digital breach differs from perceived workload inequality or general organisational unfairness. Justice frameworks evaluate the allocation of outcomes and the procedures by which they are determined (Conway and Briner, 2005). A digital breach refers to the relational interpretation of whether an implicit obligation, often unspoken by either party, has been honored or violated. Finally, digital breach differs from traditional psychological contract breach (Morrison and Robinson, 1997) through the five characteristics elaborated above. Diffuse temporality, implicit formation, technological mediation, normalised pervasiveness, and continuous negotiation render digital breach phenomenologically distinct. It requires conceptual treatment as a theoretically limited extension of traditional breach logic, rather than a straightforward application.

#### 4.2.2 Escalation from breach to violation

Consistent with Morrison and Robinson’s (1997) breach-violation distinction, escalation from digital breach to affective violation is not automatic. The probability of violation is amplified by three conditions: attributing connectivity demands to organisational indifference rather than situational necessity; high prior relational investment in the employment relationship; and cumulative breach magnitude exceeding an employee’s personal threshold of tolerable discrepancy (Tomprou et al., 2015). Where a digital breach is interpreted as contextually necessary or temporary, cognitive readjustment is more likely to occur. Identical patterns of digital availability demands may result in violation responses in some employees while creating disappointment or expectation readjustment in others. This difference, according to the model, is due to variations in attribution, relational investment, and cumulative breach historical experience.

*Proposition 2*: The positive relationship between digital breach and affective psychological contract violation is moderated by attribution and cumulative breach magnitude. Specifically, the relationship is stronger when employees attribute availability demands to organisational indifference and when the cumulative breach magnitude exceeds personal tolerance thresholds.

### 4.3 Organisational trust and relational outcome

According to Morrison and Robinson (1997), psychological contract research distinguishes between cognitive recognition of breach and emotional experiences of violation. In digitally intensive work environments, persistent connectivity demands may elicit similar affective responses, particularly if employees believe the organisation has indirectly altered the terms of exchange without mutual negotiation. These reactions may be intensified in environments characterised by high performance visibility or competitive standards.

A central relational outcome in this model is organisational trust. Trust constitutes a central relational currency in employment relationships. It is a willingness to accept vulnerability based on positive expectations regarding organisational intentions and behaviour. Dirks and Ferrin’s (2002) meta-analytic treatment of trust in leadership captures core relational dynamics of the construct, although their findings concern interpersonal trust in leadership specifically. The dimensional specification employed in this study follows Mayer et al.’s (1995) integrative model of organisational trust, which distinguishes integrity, benevolence, and ability as separable dimensions. Integrity-based trust refers to employees’ assessment of whether the organisation adheres to acceptable implicit principles. Benevolence-based trust refers to employees’ evaluation of whether the organisation demonstrates genuine concern for their interests beyond its own instrumental aims. Meta-analytic evidence confirms that these dimensions of trust carry distinct predictive relationships with risk-taking and performance behaviours, emphasising the importance of specifying which trust dimension a theoretical model implicates (Colquitt et al., 2007).

Digital breach indicates that the organisation has disregarded the implicit temporal boundaries on which employees depend for recovery and autonomy. This is most likely to erode integrity-based and benevolence-based trust rather than ability-based trust. Outcomes such as commitment or turnover intention are important downstream consequences but typically flow from broken trust (Robinson, 1996). Focusing on trust allows the model to capture the immediate relational damage of perceived violation before these downstream effects unfold. The trust outcome is articulated through two propositions, namely a direct effect (P3a) and a mediation pathway (P3b).

*Proposition 3a*: Affective psychological contract violation is negatively associated with organisational trust, particularly its integrity-based and benevolence-based dimensions.

*Proposition 3b*: Affective psychological contract violation mediates the relationship between digital breach and the erosion of organisational trust.

### 4.4 Moderating conditions

The strength of these relationships is not consistent. The mechanisms underlying the moderating effects are grounded in appraisal theory and social learning theory. Two distinct moderating conditions are proposed, each operating through a different theoretical mechanism.

*Proposition 4a*: Employee autonomy in boundary management moderates the positive relationship between connectivity expectations and digital breach. Higher perceived autonomy, operationalised as the ability to disengage from digital platforms without reputational or material penalty, weakens this path by enabling employees to cognitively frame connectivity expectations as negotiable preferences rather than obligatory demands (Barber and Santuzzi, 2015; Kossek et al., 2012, 2023).

When employees perceive genuine discretion in their availability, connectivity expectations are more likely to be viewed as flexible rather than coercive. This appraisal shift reduces the likelihood that unmet expectations will be interpreted as relational violations. The mechanism is cognitive reframing on an individual level. Autonomy redefines what the expectation means, rather than just how demanding it feels.

*Proposition 4b*: Leadership models based on limited availability norms moderate the positive relationship between connectivity expectations and digital breaches. Leaders who establish normative reference points that raise the threshold at which deviations are perceived as obligatory weaken this path by visibly segmenting their own temporal boundaries. According to research on workplace segmentation norms, employees who perceive organisational practices that support time boundaries feel less pressured to engage in work-related communication outside of contractual hours (Bandura, 1977; Derks et al., 2014).

Leadership models operate through social norm internalisation rather than individual appraisal. When leaders clearly refrain from after-hours communication, they reset employees’ baseline assumptions about expected responsiveness (Bandura, 1977), lowering the relational salience of connectivity requirements. Research on constant connectivity and boundary management in post-pandemic hybrid work confirms that informal social norms, particularly those imposed by managers, are among the strongest contextual determinants of how employees manage their digital availability boundaries (Van Zoonen et al., 2023). In contrast, leadership that demonstrates constant responsiveness strengthens connectivity expectations and intensifies the breach experience. This mechanism is social and normative. It is fundamentally different from the cognitive-appraisal pathway of P4a.

### 4.5 Organisational and contextual antecedents

P5 serves as the model’s antecedent specification rather than its primary originality claim. It identifies the organisational conditions that give rise to the connectivity expectations entering the main causal pathway. Its function is architectural. It grounds the model in the organisational conditions that produce the implicit contractual terms whose perceived violation the model seeks to explain.

The development of connectivity expectations requires both normative compliance and social information processing (Mazmanian et al., 2013). Employees actively scan their workplace for cues on what responsive, committed, and professional behaviour looks like. Leadership behaviour is the most direct and influential signal in this process (Rousseau et al., 2018). Organisational cultures that reward quick responses, whether through performance evaluations, informal recognition, or the implicit appreciation of availability, encode connectivity as an essential component of professional identity. This enhances its contractual significance. Cultures that model restricted availability, on the other hand, reduce the perceived obligatory nature of connectivity demands.

Industry characteristics and occupational roles influence how connectivity requirements are interpreted. In knowledge-intensive or globally distributed organisations, responsiveness may be framed as critical to collaboration and innovation. These dynamics remain unresolved by the adoption of new working strategies, such as remote and hybrid arrangements. Rather, it has introduced new forms of digital presence pressure that reframe availability expectations in terms of platform visibility and response delays, rather than physically shared space (Afota et al., 2023; Alfes et al., 2022). Research on telecommuting and flexible work arrangements suggests that managing availability expectations is especially important in contexts where work boundaries are already permeable by design. Unclear norms in such settings increase role ambiguity and reduce the predictability of mutual obligations (Allen et al., 2015). When expectations are inconsistent or poorly articulated, employees may be uncertain about acceptable availability levels, increasing the likelihood of a perceived breach.

*Proposition 5*: Organisational communication culture and leadership signaling are positively related to the strength and rigidity of perceived connectivity expectations.

### 4.6 Scope and boundary conditions

The DPCM operates within a specific theoretical domain and is limited by conditions under which its propositions cannot be expected to hold.

The DPCM is theorised to apply in the following contexts. First, in knowledge-intensive employment settings where work tasks are discretionary in content and timing, digital communication is the primary medium of coordination, and professional identity is partly constituted through responsiveness signals. Second, in employment relationships characterised by some degree of relational rather than purely transactional contracting, where reciprocity and implicit obligations are operative. Third, in contexts where availability norms are formed through implicit social inference rather than algorithmic dictation. Fourth, in remote, hybrid, and digitally mediated work arrangements where temporal boundaries are negotiable rather than fixed by physical presence.

The DPCM is not theorised to apply in the following contexts. First, in low-connectivity manual or service work where digital communication is peripheral to job execution and after-hours availability is not a meaningful expectation. Second, in algorithmically managed gig and platform work where availability is structurally enforced through scheduling algorithms, rating systems, and automated penalties. In such contexts, the psychological contract operates under compliance logic rather than relational reciprocity, and the breach-violation-trust pathway requires reformulation beyond the present framework. Third, in highly precarious low-autonomy roles where the psychological contract is dominated by compliance requirements. Fourth, in contexts where formal regulation, such as right-to-disconnect legislation, has substantially replaced implicit availability norms with explicit contractual terms.

## 5 Discussion

### 5.1 Reframing connectivity as a relational work design issue

The DPCM offers a reframing of constant connectivity. Rather than viewing it solely as a job demand or a boundary challenge, the framework positions it as a relational signal that shapes how employees interpret the fairness and reciprocity of the employment relationship. This relational perspective extends existing research on digital work intensification by adding a layer of meaning-making to the analysis of strain and well-being.

The model is based on assumptions that should be acknowledged. It assumes that employees interpret connectivity expectations through a relational lens rather than purely a workload lens, and that these norms are sufficiently salient to become part of the psychological contract. These assumptions are plausible within the scope conditions specified in Section 4.6 but do not extend to the boundary conditions specified there. In precarious or low-autonomy roles, in algorithmically managed work, and in contexts dominated by compliance rather than reciprocity, the framework’s relational architecture does not hold. The model’s applicability is therefore intentionally restricted.

The role of power, hierarchy, and organisational control warrants explicit attention. Connectivity expectations are not merely relational misunderstandings arising from poorly negotiated psychological contracts. They can function as instruments of organisational control and performance surveillance. Mazmanian et al.’s (2013) account of the autonomy paradox is instructive given that knowledge professionals may voluntarily reproduce connectivity expectations that ultimately constrain their own autonomy. The current model acknowledges that availability norms may serve disciplinary rather than relational purposes. In contexts where performance management systems incorporate response-time metrics or digital engagement indicators, connectivity expectations may operate as temporal disciplinary mechanisms, particularly as gig economy and platform-based work expand the scope of digitally surveilled employment (Vallas and Schor, 2020). In such cases, the psychological contract may be perceived as an imposed compliance structure rather than a negotiated exchange, fundamentally altering the breach-violation-trust process described in the model.

### 5.2 Theoretical contribution to psychological contract scholarship

The contribution of this article is conceptual and structural rather than empirical or causal. The DPCM offers a structured vocabulary, a documented synthesis of fragmented literatures, and a propositional architecture that future empirical research may refine, qualify, or revise. Specifically, the model contributes to psychological contract theory by introducing digital breach as a theoretically bounded domain of implicit relational obligation, by articulating the discrepancy architecture that links connectivity expectations to breach intensity, and by separating the direct trust effect (P3a) from the mediation pathway (P3b). It contributes to digital work research by providing a relational vocabulary that supplements, rather than replaces, technostress and JD–R perspectives. It contributes to boundary management research by linking boundary permeability to relational rather than purely tactical outcomes. The framework should be viewed as dynamic, rather than static. Rousseau et al. (2018) describe contract maintenance and disruption processes that allow for revisions to connectivity expectations and contractual salience. Digital breach may therefore trigger contract renegotiation and repair rather than irreversible trust erosion. This dynamic dimension offers a productive avenue for longitudinal empirical inquiry.

### 5.3 Implications for Understanding Employee Well-being in Digital Contexts

The DPCM has implications for research on employee well-being. Technostress and job demands perspectives emphasise the strain associated with excessive connectivity. According to the DPCM, relational interpretations may either amplify or mitigate these effects. Employees who perceive connectivity expectations as mutually negotiated may experience digital engagement as a form of flexibility or professional autonomy. When expectations are unclear or perceived as unfair, however, they may contribute to feelings of violation that intensify stress responses and reduce organisational trust. This emphasises the importance of examining well-being outcomes not only in terms of workload or technological exposure but also in relation to how organisational norms shape employees’ sense of relational justice. By situating digital work practices within the broader psychological contract, the study highlights the interconnectedness of work design, organisational communication, and employee well-being. Employees who experience digital breach may experience adverse well-being outcomes not primarily due to overload, though overload may co-occur, but because the relational foundation of their employment has been compromised. This distinction is particularly important in hybrid and remote work contexts, where technostress and boundary permeability are prevalent but may be mediated or moderated by perceived organisational support and relational fairness rather than load reduction alone (Harunavamwe and Ward, 2022; Singh et al., 2022). The model’s trust outcome (P3a) has particular significance for occupational health research. Organisational trust is a well-established antecedent of employee well-being, with low trust consistently associated with heightened emotional exhaustion, reduced affective commitment, and elevated turnover intention (Robinson, 1996). Research within the JD–R tradition provides further grounding here: Hakanen et al. (2008) demonstrated in a longitudinal study that job demands lead to burnout while job resources predict work engagement, with the quality of employment relationships functioning as a significant resource. The DPCM suggests that connectivity demands may harm well-being through two distinct pathways: overload and recovery disruption (the technostress and JD–R pathway) and cumulative loss of relational trust (the DPCM pathway), by framing digital breach as a relational mechanism that erodes integrity-based and benevolence-based trust (Mayer et al., 1995). Organisations addressing well-being exclusively through connectivity-reduction measures may achieve workload relief without restoring the relational conditions necessary for sustained well-being.

### 5.4 Practical implications for organisational communication and leadership

The model identifies two primary intervention levers through which organisations can reduce digital breach risk. The first, corresponding to P4a, is the deliberate enhancement of employee autonomy in digital boundary management. Operationally, this refers to developing conditions in which employees can disengage from platforms without reputational or material penalty. This could include explicit organisational statements that after-hours non-response is acceptable, performance evaluation criteria that do not reward continuous availability, and management practices that do not impose informal penalties for disconnection. Research on work-life flexibility policies confirms that autonomy and boundary control are critical mechanisms for converting such policies into well-being gains (Kossek et al., 2023). The second lever, which corresponds to P4b, is intentional leadership norm modelling. Leaders influence employees’ baseline expectations about required responsiveness through their own observable behaviour. Leadership that demonstrates limited availability adjusts these expectations in ways that reduce the breach-generating potential of organisational connectivity norms (Bandura, 1977). Practically, this involves leaders refraining from after-hours messaging, communicating clearly about their own digital boundaries, and supporting formal availability guidelines. The DPCM directly tests whether such interventions reduce digital breach perceptions and strengthen organisational trust. The model implies that addressing connectivity expectations is not solely a matter of workload management, it is a relational process requiring intentional governance. Organisations that treat digital availability as a purely operational concern, managing it through notification silencing or out-of-office automation alone, may achieve surface-level relief without resolving the underlying contractual dynamics the DPCM identifies.

### 5.5 Directions for future research

The conceptual nature of the current study suggests several directions for future empirical research. Longitudinal research could examine how connectivity expectations evolve over time and how perceptions of digital breach influence trust, commitment, and performance outcomes. Comparative studies across occupational groups or cultural contexts may shed light on how organisational norms and societal expectations influence interpretations of availability (Ollier-Malaterre and Foucreault, 2017). Mixed-method designs that combine survey and qualitative approaches may provide more insight into how employees negotiate connectivity expectations in practice and what attribution processes determine whether a breach progresses to a violation.

Future research should also explore the DPCM’s boundary conditions in algorithmically managed work contexts, where availability monitoring is structural and automated rather than relational and negotiated. In such contexts, the implicit psychological contract may be less a product of social inference than of algorithmic legibility, and the breach-violation-trust pathway is likely to take a fundamentally different form. Cross-cultural research should look into whether connectivity obligations follow different relational logics in high-context or collectivist work environments, where the line between professional and personal availability may be blurred (Ollier-Malaterre and Foucreault, 2017). Investigations into power imbalances between managers and employees are additionally justified, given the model’s assumption of a relational rather than compliance-oriented psychological contract. The unique dynamics of remote and hybrid work arrangements, which have significantly altered availability expectations since 2020, provide an especially pressing empirical context for testing the DPCM’s propositions (Afota et al., 2023; Alfes et al., 2022).

Future empirical work should prioritize the development and validation of a multi-item measure of digital breach intensity. A future instrument could assess: (1) the perceived obligation to respond to work communications outside of formal working hours; (2) the observed distinction between expected and actual response-time norms; (3) the perceived reputational or material risk associated with technological disengagement; and (4) a cumulative sense that availability expectations exceed what was implicitly agreed upon at employment entry. These indicator domains reflect the construct’s five theoretical characteristics, serving as a conceptual foundation for scale development and empirical testing of the DPCM’s dimensions.

## 6 Conclusion

The digitalisation of work has fundamentally redefined the temporal boundaries of employment. New expectations for employee availability, responsiveness, and professional engagement have emerged, many of which are implicit. Existing research has documented the strain that comes with constant connectivity. Less theoretical attention has been dedicated to the relational and contractual interpretations of such expectations. This article addresses that gap. It redefines connectivity expectations as an implicit domain of psychological contract obligation. Additionally, the article introduces the DPCM as a theoretical framework for investigating how digitally mediated work practices shape perceptions of reciprocity, breach, and organisational trust.

The concept of a digital breach emphasizes how differences between expected and enacted availability behaviours may provoke affective responses that influence relational attitudes toward the organisation. Existing technostress, JD–R, and boundary management frameworks are unable to adequately account for these responses. The DPCM extends psychological contract theory to temporally flexible work environments, introduce theoretically specified moderating conditions, and highlights the overlooked role of power and control in shaping connectivity expectations.

The study ultimately argues that sustainable digital work design requires technical solutions and the intentional governance of mutual expectations. Managing connectivity only as a workload challenge is insufficient. Cultivating it as a matter of relational integrity is a more defensible approach. The DPCM provides a theoretical foundation for future empirical research. Its empirical development through longitudinal designs, cross-cultural studies, and construct validation will be critical to advancing understanding of how employment relationships are constituted and contested in an age of pervasive digital connectivity.

## Data Availability

This is a conceptual study that utilises data that is publicly accessible from published literature. The data sources utilised in the study are available through the relevant journals, repositories, or databases referenced in the manuscript.
