# Supplementary figures and images for: Connectivity expectations as psychological contract terms in the digital workplace
Source: Front Psychol. 2026 Jun 4;17:1852486. doi: 10.3389/fpsyg.2026.1852486 (PMC13275278; doi:10.3389/fpsyg.2026.1852486)

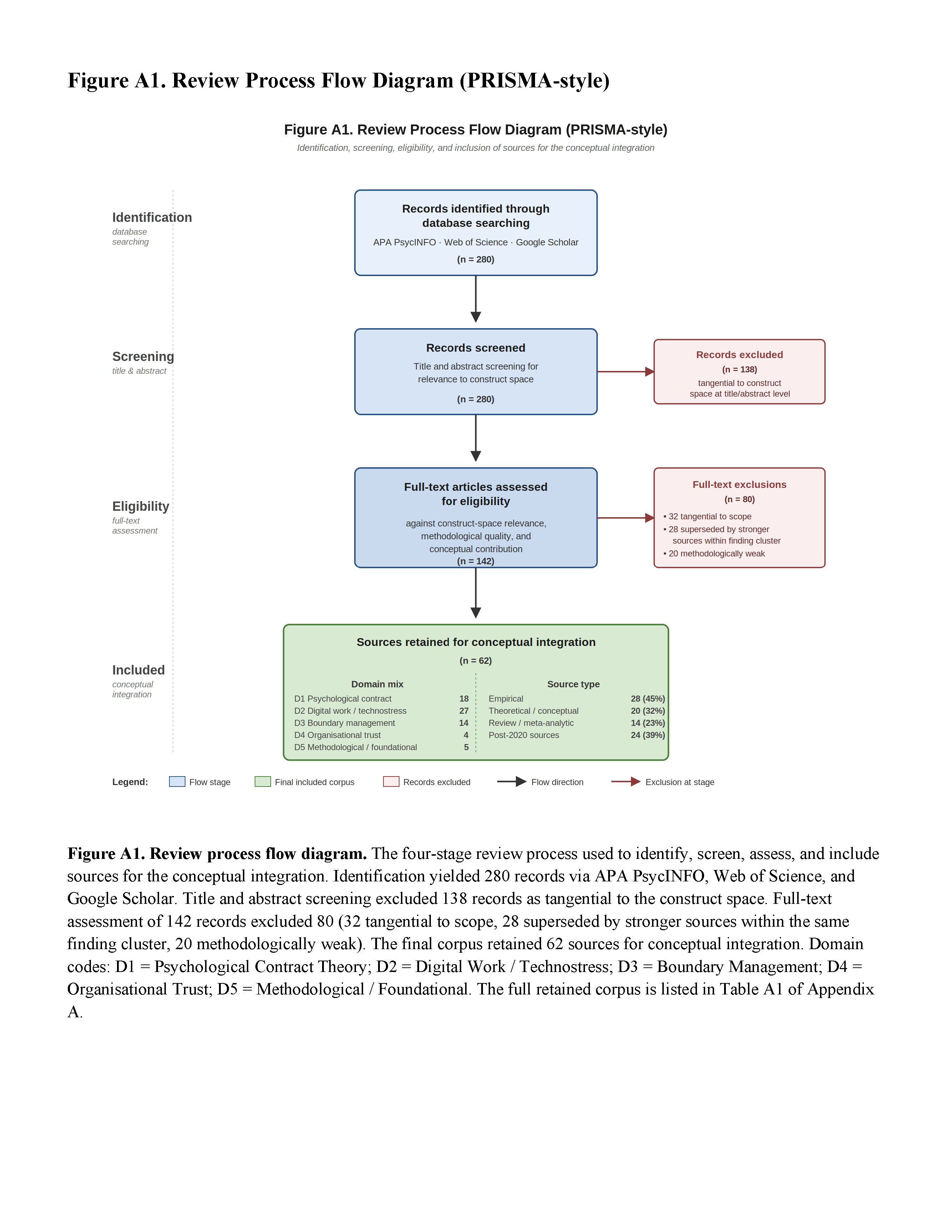

Supplement: Supplementary file 1 [file Image_1.TIFF]
