# Supplementary material for: Connectivity expectations as psychological contract terms in the digital workplace
Source: Front Psychol. 2026 Jun 4;17:1852486. doi: 10.3389/fpsyg.2026.1852486 (PMC13275278; doi:10.3389/fpsyg.2026.1852486)
Supplement: Supplementary file 2 [file Supplementary_File_1.docx]

**Appendix A. Literature Corpus and Review Process**

This appendix provides full transparency on the literature base supporting the Digital Psychological Contract Model (DPCM). Section A.1 describes the corpus identification and screening procedure. Section A.2 reports corpus composition. Table A1 lists all sources with domain coding, type coding, and the model components each source informs. Figure A1 depicts the review process flow.

**A.1 Identification and Screening Procedure**

Literature was identified through structured purposive searching across three core theoretical domains: psychological contract theory (D1), digital work and technostress (D2), and boundary management (D3). Two cross-cutting domains, organisational trust (D4) and methodological/foundational sources (D5), were retained for theoretical anchoring. Search platforms were APA PsycINFO, Web of Science, and Google Scholar. Search terms included: “psychological contract,” “psychological contract breach,” “psychological contract violation,” “digital work,” “after-hours communication,” “technostress,” “boundary management,” “work-home boundary,” “remote work,” “hybrid work,” and “always-on.”

The search was carried out between June and October 2024. The initial retrieval yielded approximately 280 records from the three primary domains. After screening the titles and abstracts for relevance to the construct space, 142 records remained. Full-text screening removed sources that were either highly tangential to the construct space, methodologically weak, or duplicated by stronger sources within the same finding cluster. The final corpus retained for the conceptual integration consists of 62 sources, listed in Table A1.

**A.2 Corpus Composition**

The 62 retained sources are divided as follows. Psychological contract theory (D1) has 18 sources; digital work and technostress (D2) has 27 sources; boundary management (D3) has 14 sources; organisational trust (D4) has 4 sources; and methodological/foundational (D5) has 5 sources. By type, there were 28 empirical studies (45%), 20 theoretical and conceptual works (32%), and 14 reviews and meta-analyses (23%). By date: 24 sources (39%) published after 2020, addressing post-pandemic digital work dynamics; 38 sources (61%) older, primarily theoretical foundations and pre-pandemic empirical evidence retained for construct grounding.

The five dimensions of digital breach outlined in Section 4.2 were derived from explicit source clusters in this corpus:

Diffuse temporality was primarily derived from Wajcman (2015), Wajcman and Rose (2011), Messenger et al. (2017), and Schlachter et al. 2018.

Mazmanian et al. (2013), Conway and Briner (2005), and Rousseau et al. (2018) all contributed to the concept of implicit formation.

Mazmanian et al. (2013), Tarafdar et al. (2019), Ayyagari et al. (2011), and Vallas and Schor (2020) all contributed to the concept of technological mediation, with Van Zoonen et al. (2023) supporting the constitutive role argument.

Mazmanian et al. (2013), Ter Hoeven et al. (2016), Alfes et al. (2022), and Afota et al. (2023) all contributed to the normalised pervasiveness concept.

Continuous negotiation is based on Rousseau et al. (2018), Tomprou et al. (2015), and the boundary-renegotiation literature (Kreiner et al., 2009; Kossek et al., 2012, 2023).

**A.3 Table A1 - Full Literature Corpus**

Table A1 lists each source in the final corpus with three coding dimensions: (1) primary theoretical domain(s); (2) source type; (3) the manuscript component informed. Domain codes: D1 = psychological contract theory; D2 = digital work and technostress; D3 = boundary management; D4 = organisational trust; D5 = methodological/foundational. Type codes: E = empirical; T = theoretical/conceptual; R = review or meta-analysis; M = methodological/foundational guideline.

| **Source** | **Domain** | **Type** | **Informs** |
| --- | --- | --- | --- |
| Afota et al. (2023) | D2 | E | Section 4.5, Section 5.1 |
| Alfes et al. (2022) | D2 | R | Section 1, Section 4.1, Section 4.5, Section 5.1 |
| Allen et al. (2015) | D3 | R | Section 4.5 |
| Ashforth et al. (2000) | D3 | T | Section3.4 |
| Ayyagari et al. (2011) | D2 | E | Section 1, Section 3.1, Section 4.2 |
| Bakker & Demerouti (2017) | D2 | T | Section 3.4 |
| Ballas et al. (2024) | D1, D2 | R | Section 1, Section 3.2, Section 5.2 |
| Bandura (1977) | D5 | T | P4b mechanism |
| Barber et al. (2019) | D2 | E | Section1 |
| Barber & Santuzzi (2015) | D3 | E | Section 3.3, P4a |
| Becker et al. (2022) | D2, D3 | E | Section 3.1, Section 4.4 |
| Blau (1964) | D5 | T | Section 3.4 |
| Butts et al. (2015) | D3 | E | Section 3.3 |
| Chesley (2005) | D3 | E | Section 3.1 |
| Clark (2000) | D3 | T | Section 3.3 |
| Colquitt et al. (2007) | D4 | R | Section 4.3 |
| Conway & Briner (2005) | D1 | R | Section 1, Section 3.2, Section 4.2, Section 5.2 |
| Coyle-Shapiro & Kessler (2000) | D1 | E | Section 3.2 |
| Cropanzano & Mitchell (2005) | D5 | R | Section 3.4 |
| Derks et al. (2014) | D2, D3 | E | Section 3.1, P4b |
| Dettmers (2017) | D2, D3 | E | Section 3.1 |
| Dirks & Ferrin (2002) | D4 | R | Section 4.3 |
| Eurofound (2021) | D2 | R | Section 1, Section 3.1 |
| Fenner & Renn (2010) | D2, D3 | E | Section 3.1 |
| Gadeyne et al. (2018) | D2, D3 | E | Section 2.1 |
| Guest (1998) | D1 | T | Section 3.2 |
| Hakanen et al. (2008) | D2 | E | Section 5.3 |
| Harunavamwe & Ward (2022) | D2 | E | Section 3.1, Section 5.3 |
| Jaakkola (2020) | D5 | M | Section 2.0, Section 2.1, Section 2.3 |
| Kossek et al. (2012) | D3 | E | Section 3.3, P4a |
| Kossek et al. (2023) | D3 | R | Section 4.4, Section5.4 |
| Kreiner et al. (2009) | D3 | E | Section 3.3 |
| Mayer et al. (1995) | D4 | T | Section 4.3 |
| Mazmanian et al. (2013) | D2, D3 | E | Section 4.1, Section 4.2, Section 4.5, Section5.1 |
| Messenger et al. (2017) | D2 | R | Section 1, Section 3.1 |
| Morrison & Robinson (1997) | D1 | T | Section 1, Section 3.2, Section 4.2, Section 4.3 |
| Ng et al. (2010) | D1 | E | Section 3.2 |
| Nippert-Eng (1996) | D3 | T | Section 3.3 |
| Ollier-Malaterre & Foucreault (2017) | D2, D3 | R | Section 2.4, Section 5.5 |
| Ragu-Nathan et al. (2008) | D2 | E | Section 3.1 |
| Robinson (1996) | D1, D4 | E | Section 4.3, Section 5.3 |
| Robinson & Morrison (2000) | D1 | E | Section 1, Section 3.2 |
| Rousseau (1989) | D1 | T | Section 3.2 |
| Rousseau (1995) | D1 | T | Section 1, Section 4.1 |
| Rousseau et al. (2018) | D1 | T | Section 1, Section 3.2, Section 4.1, Section 4.5, Section 5.2 |
| Schaufeli & Bakker (2004) | D2 | E | Section 3.4 |
| Schlachter et al. (2018) | D2, D3 | R | Section 2.1, Section 3.1 |
| Singh et al. (2022) | D2 | E | Section 1, Section 3.1 |
| Sonnentag (2012) | D2 | R | Section 1, Section 3.1 |
| Sonnentag & Fritz (2015) | D2 | R | Section 1, Section 3.1 |
| Tarafdar et al. (2019) | D2 | R | Section 1, Section 3.1, Section 4.2 |
| Ter Hoeven et al. (2016) | D2 | E | Section 3.1 |
| Tomprou et al. (2015) | D1 | T | Section 4.2 |
| Topa et al. (2022) | D1 | R | Section 1, Section 3.2, Section 5.2 |
| Towers et al. (2006) | D2, D3 | E | Section 3.1 |
| Vallas & Schor (2020) | D2 | R | Section 5.1 |
| Van Zoonen et al. (2023) | D2, D3 | E | Section 1, Section 3.1, Section 3.3, Section 4.4 |
| Wajcman (2015) | D2 | T | Section 3.1 |
| Wajcman & Rose (2011) | D2 | T | Section 1 |
| Whetten (1989) | D5 | M | Section 2.3 |
| Zhang et al. (2023) | D1, D2 | E | Section 1, Section 3.2, Section 5.2 |
| Zhao et al. (2007) | D1 | R | Section 1, Section 3.2 |

*Note. Codes - Domain: D1 = Psychological Contract Theory; D2 = Digital Work / Technostress; D3 = Boundary Management; D4 = Organisational Trust; D5 = Methodological / Foundational. Type: E = Empirical; T = Theoretical/Conceptual; R = Review/Meta-analysis; M = Methodological/Foundational.*

**A.4 Figure A1 - Review Process Flow**

Figure A1 (separate file) depicts the four-stage review process: identification, screening, eligibility, inclusion. Source counts at each stage are: identified (n = 280); screened by title and abstract (n = 280); excluded at title/abstract (n = 138); full-text eligibility assessment (n = 142); excluded at full-text (n = 80, including 32 tangential to construct space, 28 superseded by stronger sources within finding clusters, and 20 methodologically weak); included in final corpus (n = 62).
